# Healthy Lifestyle Behaviors Attenuate the Effect of Poor Sleep Patterns on Chronic Kidney Disease Risk: A Prospective Study from the UK Biobank

**DOI:** 10.3390/nu16234238

**Published:** 2024-12-08

**Authors:** Xia Lin, Jiali Lv, Shuai Zhang, Xiaoyan Ma, Xiaofeng Zhang, Cheng Wang, Tao Zhang

**Affiliations:** 1 Department of Biostatistics, School of Public Health, Cheeloo College of Medicine, Shandong University, Jinan 250012, China; 2 Institute for Medical Dataology, Cheeloo College of Medicine, Shandong University, Jinan 250002, China

**Keywords:** chronic kidney disease, lifestyles, sleep patterns, prospective cohort study

## Abstract

Objectives: This study aimed to assess the impact of modifiable lifestyle behaviors on the association between sleep patterns and chronic kidney disease (CKD) risk. Methods: This study included 294,215 UK Biobank participants initially without CKD, followed until 13 October 2023. Sleep patterns were derived from five sleep factors, including sleep duration, chronotype, insomnia, snoring, and daytime dozing. The healthy lifestyle score (HLS) was newly calculated based on smoking status, physical activity, diet, body mass index, and mental health. Cox’s proportional hazards models were used to assess the associations between sleep patterns, HLS, and CKD risk. Results: A total of 17,357 incident CKD cases were identified during a median follow-up of 14.5 (interquartile range: 13.7–15.3) years. Both sleep patterns and HLS were independently associated with increased CKD risk (*p*-trend < 0.001). Importantly, the HLS was found to modify the association between sleep patterns and CKD risk (*p*-interaction = 0.026). Among participants with a low HLS, medium (HR = 1.12; 95% CI 1.05–1.19) and poor sleep patterns (HR = 1.23; 95% CI 1.17–1.30) increased CKD risk to varying degrees, whereas no significant association was observed for a high HLS. Moreover, the combination of a low HLS and poor sleep pattern significantly increased the risk of incident CKD (HR = 2.19; 95% CI 2.00–2.40). Conclusions: A high HLS may significantly reduce CKD risk associated with poor sleep, whereas a low HLS may exacerbate this risk. These findings underscore the critical importance of lifestyle interventions as a primary prevention strategy for CKD.

## 1. Introduction

Chronic kidney disease (CKD) is a progressive disease that affects approximately 10% of the adult population worldwide [1]. Since CKD often presents no early symptoms and is often diagnosed too late to slow its progression, the early prevention of CKD has critical public health significance for alleviating the disease burden [2,3]. Fortunately, in considering the substantial plasticity of renal function, the early identification of modifiable risk factors for CKD can profoundly prevent adverse outcomes [4].

Previous studies have demonstrated associations between individual sleep factors and CKD development, such as sleep duration [5,6], chronotype [7], snoring [8], insomnia [9], and daytime dozing [10]. These sleep-related factors are not isolated, and they often interact with one another, potentially influencing CKD risk [11]. Maintaining a healthy sleep pattern is inversely associated with CKD risk, while a worse sleep pattern is implicated in a heightened risk of CKD onset [12,13]. Moreover, studies have shown a close relationship between sleep factors and individuals’ lifestyle behaviors. Participants with poor sleep factors are more likely to exhibit unhealthy lifestyle behaviors [14]. Traditional lifestyle behaviors, including smoking status [15,16], physical activity [17,18], diet [19,20,21], and body mass index (BMI) [22,23] have been identified as contributors to CKD risk. Mental health has been widely proven to be an important risk factor for CKD onset, yet the existing healthy lifestyle score (HLS) often fails to consider this crucial factor [24,25,26]. Moreover, most studies have primarily explored the association between a healthy lifestyle and CKD prognosis, with limited research examining how such a lifestyle impacts the risk of developing CKD among the general population [27,28,29].

Therefore, based on a UK Biobank study, we constructed a sleep pattern score by integrating five distinct sleep factors and formulated a healthy lifestyle score through the integration of several lifestyle behaviors. Our study aimed to examine the independent associations between sleep patterns, HLS, and CKD risk and to explore whether adherence to an overall healthy lifestyle modifies the CKD risk associated with sleep patterns. Additionally, we evaluated the combined effect of sleep patterns and HLS on CKD risk, accounting for variability in outcomes across different demographic groups. Ultimately, we found that HLS can modify the associations between sleep patterns and CKD risk, providing new insights into the risk management of CKD.

## 2. Methods

### 2.1. Study Population

**Figure 1:**
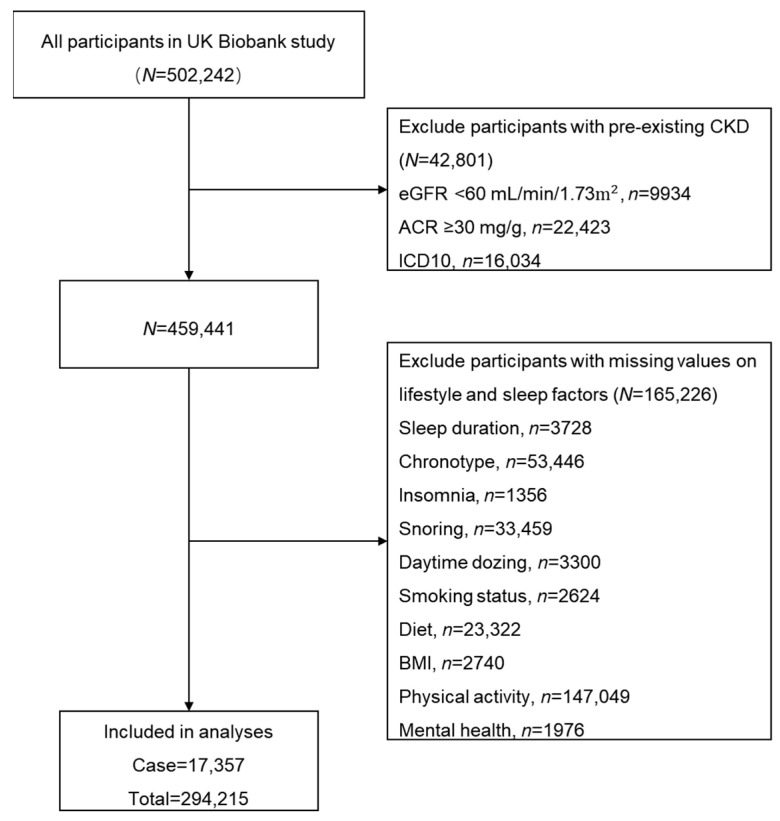
Study flow diagram. ACR = albumin–creatinine ratio; BMl = body mass index; CKD = chronic kidney disease; eGFR = estimated glomerular filtration rate; ICD10 = the International Classification of Diseases, 10th revision.

The UK Biobank, a large-scale prospective cohort study, enrolled approximately 500,000 participants aged 37 to 73 years from 22 assessment centers across the United Kingdom during the period from 2006 to 2010 [30,31]. The participants completed a comprehensive questionnaire and physical measurements at baseline. Figure 1 illustrates the selection process for the study participants. Preliminary analyses excluded participants diagnosed with CKD at baseline (*n* = 42,801) and those with missing data on lifestyle behaviors or sleep factors (*n* = 165,226). Ultimately, a total of 294,215 participants were included in subsequent analyses.

### 2.2. Ascertainment of Chronic Kidney Disease

Incident CKD cases were defined by the International Classification of Diseases, 10th revision (ICD-10) codes N03, N06, N08, N11-N16, and N18-N21 (Supplemental Table S1) after baseline assessment. Pre-existing CKD cases were identified using any of the following criteria: (1) the ICD-10 codes abovementioned; (2) an estimated glomerular filtration rate (eGFR) less than 60 mL/min/1.73 m^2^; and (3) an albumin–creatinine ratio (ACR) ≥ 30 mg/g [32]. The eGFR estimation was performed using the Chronic Kidney Disease Epidemiology Collaboration (CKD-EPI) equation, and serum creatinine levels were measured using enzymatic analysis on a Beckman Coulter AU5800 analyzer, as described in the UK Biobank [11,33].

### 2.3. Covariates

Information about sociodemographic characteristics was obtained from a baseline touchscreen questionnaire, including age, sex, ethnicity (white or nonwhite), Townsend deprivation index (TDI: low, intermediate, or high), education (higher degree, any school degree, vocational qualifications, or unknown), household income (less than 51,999, greater than 52,000, or unknown), hypertension (yes or no) and diabetes (yes or no). The TDI was reclassified into three categories based on the tertiles. Hypertension was determined from the average of two blood pressure readings (average systolic blood pressure ≥ 140 mmHg or average diastolic blood pressure ≥ 90 mmHg) or self-reported diagnosis or intake of antihypertensives. Diabetes was defined by a glycated hemoglobin (HbA1c) level greater than 6.5%, a self-reported diagnosis, or insulin use [34]. Further information on covariates is available in the Supplemental Methods section of the Supplementary Materials. The definitions of the sleep pattern and HLS components are provided in Supplemental Tables S2 and S3, respectively.

### 2.4. Sleep Patterns

All sleep factors were self-reported via a standardized touchscreen questionnaire at baseline. Our study’s sleep patterns were composed of five distinct sleep factors, and the poor sleep factors were defined as sleep duration <7 h/day or >8 h/day, evening chronotype (“evening” or “more evening than morning”), insomnia, snoring, and daytime dozing (Supplemental Table S2) [11,35,36,37,38]. The β coefficients of each sleep factor obtained from the Cox regression were used to construct a weighted sleep score (Supplemental Table S3). Weighted sleep score calculation: (β_sleep duration_ × sleep duration + β_chronotype_ × chronotype + β_insomnia_ × insomnia + β_snoring_ × snoring + β_daytime dozing_ × daytime dozing) × (5/sum of the β-coefficients) [35]. Finally, we categorized sleep patterns into three groups: healthy sleep pattern (4–5 points), intermediate sleep pattern (2–3 points), and poor sleep pattern (0–1 points).

### 2.5. Healthy Lifestyle Score

The healthy lifestyle score (HLS) was based on five CKD risk factors, including physical activity, smoking status, diet, BMI, and mental health (Supplemental Table S4) [39,40,41,42,43,44]. A weighted HLS was calculated as (β_physical activity_ × physical activity + β_smoking status_ × smoking status + β_diet_× diet + β_BMI_× BMI + β_mental health_ × mental health) × (5/sum of the β-coefficients) [45] (Supplemental Table S5). Finally, the HLS was categorized into three levels: high HLS (4–5 points), medium HLS (2–3 points), and low HLS (0–1 points).

### 2.6. Statistical Analysis

The participants’ follow-up years were calculated from the date of attending the assessment center to the date of CKD diagnosis, death, or the end of follow-up (13 October 2023), whichever came first. All continuous variables are described as medians and interquartile ranges (IQRs), and categorical variables are summarized using frequencies and percentages.

We used multivariate-adjusted Cox proportional hazards models to calculate hazard ratios (HRs) and 95% confidence intervals (CIs) to explore the association between sleep patterns and HLS on CKD risk. The proportional hazards assumption was tested with the Schoenfeld residual method and satisfied. Two scales (multiplication and additive interactions) were used to investigate whether the risk of sleep patterns on CKD was modified by the HLS. The multiplication interactions were assessed by adding a cross-product term to the fully adjusted model. The additive interactions were investigated by estimating the relative excess risk due to interaction (RERI) and attributable proportion due to interaction (AP).

Additionally, the joint associations were explored by categorizing participants into nine joint groups (obtained by multiplying three sleep patterns and three HLS). Participants with a high HLS and healthy sleep pattern were set as the reference group. The multivariate-adjusted models accounted for covariates, including age, sex, ethnicity, TDI, education, household income, hypertension, and diabetes. There were no missing data for covariates other than TDI, education, and household income. Unknown indicators were used for missing values in categorical covariates. Furthermore, we also calculated population attributable fractions (PAFs) to estimate the proportion of CKD cases that could be attributed to modifiable lifestyle behaviors or sleep factors.

Several sensitivity analyses were performed to minimize the risk of reverse causality and evaluate the robustness of the results. First, participants who developed CKD within the first two years of follow-up were excluded. Second, weighted sleep patterns were reconstructed by excluding nonsignificant sleep factors after multivariate adjustment, and the main analyses were repeated. Third, dyslipidemia was included as an additional covariate in the analysis. All the statistical analyses were performed using R, version 4.3.1, and a two-sided *p* < 0.05 was considered to indicate statistical significance.

## 3. Results

### 3.1. Baseline Characteristics of Participants

Table 1 presents the baseline characteristics of the study population by sleep patterns. Among the 294,215 participants (median age: 57.0 [IQR: 49.0–63.0] years; 53.2% female; 91.2% white ethnicity), 17,357 (5.9%) developed CKD during a median follow-up of 14.5 [IQR: 13.7–15.3] years. The proportions of new-onset CKD for healthy sleep pattern, intermediate sleep pattern, and poor sleep pattern were 5.3%, 6.6%, and 7.2%, respectively. Participants with a poor sleep pattern tended to be older, be male, have higher TDI, have lower levels of education, and have lower household incomes. Notably, unhealthy lifestyle behaviors were more common among participants with poor sleep pattern, while those with healthy sleep patterns had a greater proportion of high and medium HLSs. To provide additional details, baseline characteristics stratified by HLS levels are presented in Supplemental Table S6.

### 3.2. Sleep Factors, Lifestyle Behaviors, and CKD Risk

We first examined the separate associations of each sleep factor and lifestyle behavior with CKD risk. Poor sleep duration (HR = 1.13; 95% CI: 1.10–1.17), insomnia (HR = 1.06; 95% CI: 1.02–1.10), and daytime dozing (HR = 1.10; 95% CI: 1.06–1.13) were each independently associated with a higher CKD risk (Supplemental Table S3). Similarly, poor BMI (BMI ≤18.5 or ≥25.0 kg/m^2^) (HR = 1.37; 95% CI: 1.32–1.42), diet (HR = 1.19; 95% CI: 1.15–1.22), mental health (HR = 1.18; 95% CI: 1.15–1.22), smoking status (HR = 1.10; 95% CI: 1.07–1.14), and physical activity (HR = 1.08; 95% CI: 1.04–1.11) were all independently linked to elevated CKD risk (Supplemental Table S5).

We then explored the associations of sleep patterns and HLS with CKD risk, and the results are shown in Table 2. After adjusting for age, sex, ethnicity, TDI, education, household income, hypertension, and diabetes, as well as making mutual adjustments for sleep patterns and HLS, we found that poor sleep pattern was significantly associated with a greater risk of CKD incidence. Specifically, compared to participants maintaining a healthy sleep pattern, the adjusted HRs for intermediate and poor sleep pattern were 1.13 (95% CI: 1.09–1.17) and 1.18 (95% CI: 1.14–1.23), respectively (*p*-value for trend < 0.001). Similarly, for HLS, participants with a medium HLS (HR = 1.44; 95% CI: 1.34–1.54) and low HLS (HR = 1.85; 95% CI: 1.73–1.98) had a greater risk of CKD than those with a high HLS (*p*-value for trend <0.001).

To further investigate the specific degree of impact of sleep patterns and lifestyle behaviors on CKD, the PAFs were calculated. Notably, mental health (5.53%; 95% CI: 4.50–6.56%) was among the top three PAF contributors, which is comparable to the contribution of healthy sleep pattern (5.53%; 95% CI: 4.29–6.77%). Adherence to an overall healthy lifestyle could prevent 33.96% (95% CI: 30.05–37.86%) of CKD cases during follow-up, underscoring the significant potential of lifestyle modifications in CKD prevention (Supplemental Table S7).

### 3.3. Modification Effect of HLS on the Association of Sleep Patterns with CKD Risk

To explore the impact of HLS on modifying the relationship between sleep patterns and CKD risk, we performed a stratification analysis of the association according to HLS levels. As shown in Table 3, for participants with medium HLS, intermediate (HR = 1.10; 95% CI: 1.04–1.16) and poor sleep pattern (HR = 1.12; 95% CI: 1.05–1.19) were associated with a 10% and 12% increased risk of CKD, respectively. For those with a low HLS, these risks increased to 17% and 23% for intermediate (HR = 1.17; 95% CI: 1.11–1.23) and poor sleep pattern (HR = 1.23; 95% CI: 1.17–1.30), respectively. Interestingly, no significant associations between sleep patterns and CKD risk were observed in participants with a high HLS (all *p*-values > 0.05). These findings suggest that an overall healthy lifestyle may mitigate the adverse effects of suboptimal sleep patterns, while an unhealthy lifestyle may exacerbate these effects.

Further HLS stratification of associations between five individual sleep factors and CKD showed no significant independent association between these factors and CKD risk in participants with a high HLS (all *p*-values > 0.05). However, as the HLS decreased, an increasing number of adverse sleep factors demonstrated significant associations with CKD risk. A significant interaction effect was observed between sleep duration and HLS (*p* for interaction = 0.022) (Supplemental Table S8). Furthermore, we examined the modification of the association between sleep patterns and CKD risk by each specific lifestyle behavior, with diet and sleep patterns exhibiting a significant interaction (*p* for interaction = 0.019) (Supplemental Table S9).

### 3.4. Joint Association of HLS and Sleep Patterns with CKD Risk

**Figure 2:**
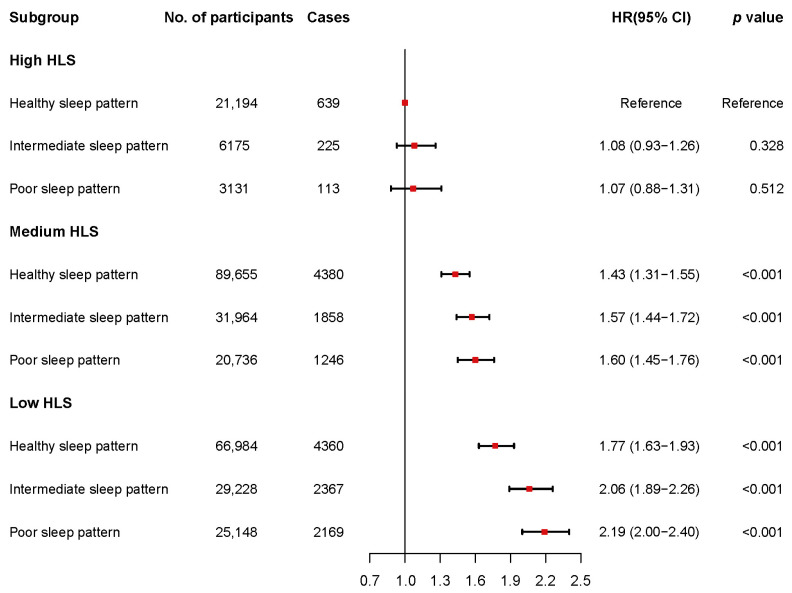
The joint association of sleep patterns and HLS with CKD risk. CKD = chronic kidney disease; HLS = healthy lifestyle score; CI = confidence interval; HR = hazard ratio.

Further analysis evaluated the combined effects of sleep patterns and HLS on CKD risk. Figure 2 illustrates a monotonic increase in CKD risk associated with the deterioration of both sleep patterns and HLS. Participants with a low HLS and poor sleep pattern had the highest CKD risk (HR = 2.19; 95% CI: 2.00–2.40) compared to those with high HLS and healthy sleep pattern. A significant interaction between sleep patterns and HLS on CKD risk was identified on the multiplicative scale (*p* for interaction = 0.026). On an additive scale, positive interactions were also observed. Specifically, individuals with a low HLS and poor sleep pattern had an RERI of 0.18 (95% CI: 0.14–0.23) and an AP of 0.11 (95% CI: 0.05–0.17). Those with a low HLS and intermediate sleep pattern had an RERI of 0.13 (95% CI: 0.09–0.18) and an AP of 0.07 (95% CI: 0.03–0.11) (Supplemental Table S10).

Kaplan–Meier (KM) curves demonstrate that individuals with a low HLS consistently exhibited a higher cumulative incidence of CKD compared to those with a medium or high HLS across all sleep pattern groups (Log-rank *p* < 0.001 for all groups) (Supplemental Figures S1–S3).

**Figure 3:**
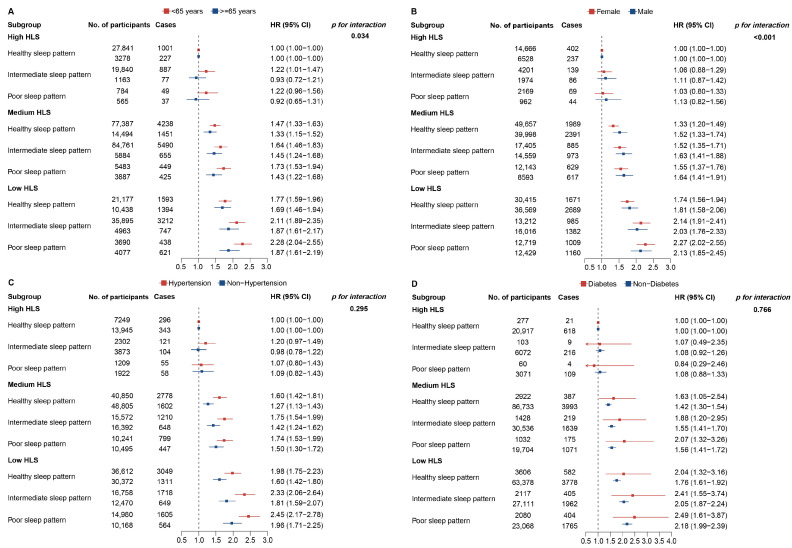
Risk of incident CKD according to sleep patterns and HLS stratified by sociodemographic variables. (**A**) Risk of incident CKD according to sleep patterns and HLS stratified by age. (**B**) Risk of incident CKD according to sleep patterns and HLS stratified by sex. (**C**) Risk of incident CKD according to sleep patterns and HLS stratified by hypertension. (**D**) Risk of incident CKD according to sleep patterns and HLS stratified by diabetes. CKD = chronic kidney disease; HLS = healthy lifestyle score; CI = confidence interval; HR = hazard ratio.

Figure 3 presents the results of the subgroup analyses. The combined effects of HLS and sleep patterns in each subgroup were consistent with the trend shown in Figure 2. In particular, the estimates of the combined effects were stronger in younger participants (<65 years old), females, and those with hypertension and diabetes. However, significant interactions with HLS and sleep patterns were observed only for age (*p* for interaction = 0.034) and sex (*p* for interaction <0.001) (Supplemental Table S11).

### 3.5. Sensitivity Analysis

According to the sensitivity analyses, the results remained robust after excluding participants who developed CKD within the initial two years of follow-up (*n* = 2719) (Supplemental Tables S12 and S13), after reconstructing weighted sleep patterns (Supplemental Tables S14–S17), and after including dyslipidemia as a covariate in the analysis (Supplemental Table S18 and Figure S4). At a high HLS, no significant association was observed between sleep patterns and CKD, and the trends in associations at a medium and low HLS aligned with the main analysis in Table 3. The estimates of the joint association slightly weakened in comparison to the main analysis in Figure 2, but the additive interactions were still confirmed. Overall, a low HLS may exacerbate the negative effects of poor sleep pattern on CKD risk, whereas a high HLS might mitigate these effects.

## 4. Discussion

In this large prospective cohort study, we developed a novel HLS that incorporates mental health alongside four traditional lifestyle behaviors. To the best of our knowledge, this is the first study to include mental health within an HLS framework. Our findings reveal that a high HLS significantly mitigated the adverse effects of poor sleep patterns on CKD risk. Conversely, individuals with a low HLS experienced a significantly increased risk of CKD associated with poor sleep pattern. This study demonstrates that the association between sleep patterns and CKD risk can be modified with a comprehensive HLS, although the underlying mechanisms of this interaction remain to be investigated.

Previous epidemiological studies have consistently demonstrated that abnormal sleep duration, chronotype, insomnia, snoring, and daytime dozing are significant risk factors for CKD [5,6,7,8,9,10]. Several of these studies have also evaluated combinations of sleep factors and have shown that individuals with poor sleep pattern face the highest risk of CKD, which is consistent with our findings [11,12]. Additionally, unhealthy sleep is associated with obesity, diabetes, and hypertension, which are established independent risk factors for CKD [46,47,48]. Other studies have also suggested that both insufficient and excessive sleep duration contribute to insulin resistance, systemic inflammation, increased sympathetic nervous system activity, and salt retention [49], all of which may lead to glomerular endothelial dysfunction and subsequent renal dysfunction.

Our study revealed that as the HLS decreased, the number of adverse sleep factors significantly associated with CKD risk increased, likely due to the complex relationship between lifestyle behaviors and sleep factors. For instance, smokers, whether current, former, or passive, often experience difficulties in sleep initiation and maintenance, increased daytime sleepiness, and poorer sleep quality [50,51]. This may be attributed to the disruptive effects of nicotine on the sleep–wake cycle and nocturnal cravings [52]. In contrast, physical activity positively influences sleep quality and serves as an effective remedy for insomnia [53]. It may extend total sleep duration by reducing insulin resistance, decreasing inflammation markers, regulating circadian rhythms, and stimulating brain-derived neurotrophic factor secretion [54]. This positive effect was echoed in dietary patterns, where a balanced intake of vegetables and fish improves sleep outcomes, contrasting sharply with the detrimental effects of the high consumption of confectionery, noodles, and generally unhealthy eating habits [55]. A systematic review has shown that healthy dietary choices promote better sleep quality by circulating intestinal hormones and stimulating the synthesis of serotonin and melatonin, whereas unhealthy dietary choices, such as processed meats and refined carbohydrates, may contribute to systemic inflammation through the release of pro-inflammatory cytokines, thereby affecting sleep [56]. Additionally, sleep disturbances are linked to physical health indicators, such as reduced abdominal subcutaneous fat, and psychological factors, such as depressive symptoms [57]. Anxiety, in particular, is often associated with increased fatigue, non-restorative sleep, and the perception of insufficient sleep [58,59]. Therefore, our findings underscore the critical need to incorporate mental health into lifestyle management strategies for CKD prevention, as individuals who regularly experience stress and anxiety are more likely to experience poorer sleep quality.

Our findings of the interactions between the HLS and sleep patterns on CKD possess biological plausibility. Physical activity may mitigate kidney damage by reducing cardiovascular disease risk factors, including blood pressure, dyslipidemia, and hyperglycemia [60,61,62]. Smoking contributes to the development of albuminuria and endothelial cell-dependent vasodilation through mechanisms such as insulin resistance [63] and endothelial cell dysfunction [64]. Moreover, glycotoxins in cigarette smoke induce the formation of advanced glycoylation end products (AGEPs), which can increase vascular permeability and promote vascular pathological changes [65]. Diet plays a crucial role; a healthy diet can prevent CKD and albuminuria, whereas poor dietary choices exacerbate inflammatory responses and oxidative stress, promoting CKD progression [19,66]. Additionally, obesity may exacerbate kidney damage through hemodynamic alterations, inflammation, and oxidative stress [67]. Evidence from a large two-sample MR study showed that a high BMI was associated with an increased risk of microalbuminuria, and the pathway from obesity to albuminuria may be mediated via type 2 diabetes (T2D) [68]. Our inclusion of mental health revealed its significant impact on CKD incidence. This association was supported by machine learning modeling and prospective follow-up studies that validated mental health as a significant predictor of CKD events and linked depressive symptoms to an increased risk of CKD and rapid kidney function decline [43,69,70]. The mechanistic pathways likely involve the activation of the hypothalamic–pituitary–adrenal axis through anxiety and depression, exacerbating inflammation and disrupting renal microcirculation, thereby inducing kidney damage [70,71].

In this study, we proposed a multifaceted hypothesis: sleep factors and lifestyle behaviors intersect to influence CKD onset through shared mechanisms, such as inflammation amplification, oxidative stress, renal blood flow modifications, sympathetic nervous system activation, and glucose metabolism alterations. Additionally, lifestyle and sleep-related factors may indirectly increase CKD risk by exacerbating conditions such as obesity, diabetes, and hypertension. The cumulative effect of lifestyle behaviors on CKD risk, supported by previous studies [72] and our PAF results, highlights the significant potential of modifiable lifestyle behaviors in reducing the CKD burden. Consequently, we advocate for a comprehensive approach that considers both lifestyle and sleep factors, aiming to adopt a holistic perspective to explore the complexities between these factors and CKD risk. This comprehensive strategy is essential for gaining insights into their interaction and designing effective interventions to decrease CKD incidence.

Additionally, our analysis of the stratified and joint associations among sleep patterns, HLS, and CKD risk revealed that a lower HLS exacerbates the association between poor sleep pattern and CKD onset, underscoring the protective benefits of a high HLS against CKD, regardless of sleep patterns. Subgroup analyses indicated a more pronounced combined effect of sleep patterns and HLS on individuals younger than 65 years old, females, and those with hypertension or diabetes, highlighting the importance of tailored interventions.

### Strengths and Limitations

The strengths of our study include the following: (1) It has a prospective design featuring a large sample size and a follow-up period exceeding that of previous studies, with a median follow-up of 14.5 years. (2) Our sleep patterns cover several key sleep factors, and we further considered mental health when constructing the HLS. (3) Unlike previous studies on patients with CKD, our study included the general population from the UK Biobank and focused on new-onset CKD outcomes. (4) Our study is among the first to investigate the interactions between sleep patterns and HLS regarding CKD risk, providing a holistic approach to CKD prevention that encompasses both sleep patterns and overall lifestyles.

Several limitations of this study need to be considered. (1) Lifestyle behaviors and sleep factors were self-reported and recorded only at baseline, which may introduce recall bias and overlook changes over time. (2) Despite comprehensive adjustments for potential confounders, we cannot entirely exclude the presence of residual confounders. (3) Despite the large sample size, our participants were mostly white, so it is unclear whether our findings can be generalized to other races. (4) Given the observational nature of this study, we cannot make conclusions about causality among HLS, sleep patterns, and CKD risk. Further randomized clinical trials are needed to confirm our findings. (5) The UK Biobank cohort primarily consists of middle-aged and older adults, limiting the generalizability of our findings to younger populations, such as teenagers, who may have different sleep factors and lifestyle behaviors.

## 5. Conclusions

In conclusion, our study provides evidence supporting the effect of an HLS on sleep patterns and CKD incidence. Our findings highlight that HLS interventions can mitigate the negative effects of poor sleep pattern and advocate a holistic approach to preventing new-onset CKD. Given lifestyle–sleep interaction patterns, healthcare providers and policymakers can develop more effective interventions and guidelines to reduce CKD incidence in the future.

## Figures and Tables

**Table 1:** Baseline characteristics of the study participants.

Characteristic	Total	Healthy Sleep Pattern	Intermediate Sleep Pattern	Poor Sleep Pattern
Participants, *n* (%)	294,215 (100.0)	177,833 (60.4)	67,367 (22.9)	49,015 (16.7)
CKD, *n* (%)	17,357 (5.9)	9379 (5.3)	4450 (6.6)	3528 (7.2)
Age, *n* (%)				
Continuous, years	57.0 [49.0; 63.0]	56.0 [49.0; 62.0]	57.0 [50.0; 63.0]	58.0 [51.0; 63.0]
<65	245,466 (83.4)	149,623 (84.1)	55,357 (82.2)	40,486 (82.6)
≥65	48,749 (16.6)	28,210 (15.9)	12,010 (17.8)	8529 (17.4)
Female, *n* (%)	156,587 (53.2)	94,738 (53.3)	34,818 (51.7)	27,031 (55.1)
White ethnicity, *n* (%)	268,305 (91.2)	162,963 (91.6)	60,692 (90.1)	44,650 (91.1)
Townsend deprivation index, *n* (%)				
Continuous	−2.3 [−3.7; 0.1]	−2.4 [−3.8; −0.2]	−2.2 [−3.7; 0.3]	−2.0 [−3.6; 0.8]
Low (Quantile 1)	104,136 (35.4)	65,884 (37.0)	22,134 (32.9)	16,118 (32.9)
Intermediate (Quantile 2)	99,813 (33.9)	61,137 (34.4)	22,378 (33.2)	16,298 (33.3)
High (Quantile 3)	89,915 (30.6)	50,608 (28.5)	22,778 (33.8)	16,529 (33.7)
Education, *n* (%)				
Higher degree	107,320 (36.5)	69,619 (39.1)	22,122 (32.8)	15,579 (31.8)
Any school degree	132,650 (45.1)	79,102 (44.5)	30,656 (45.5)	22,892 (46.7)
Vocational qualifications	14,842 (5.0)	8755 (4.9)	3562 (5.3)	2525 (5.2)
Household income less than 51,999, *n* (%)	185,417 (63.0)	108,515 (61.0)	44,233 (65.7)	32,669 (66.7)
Diabetes, *n* (%)	13,625 (4.6)	6805 (3.8)	3648 (5.4)	3172 (6.5)
Hypertension, *n* (%)	145,773 (49.5)	84,711 (47.6)	34,632 (51.4)	26,430 (53.9)
Current or previous smoking, *n* (%)	131,769 (44.8)	76,078 (42.8)	30,849 (45.8)	24,842 (50.7)
BMI, *n* (%)				
Continuous, kg/m^2^	26.5 [24.0; 29.5]	26.2 [23.8; 29.1]	26.8 [24.2; 29.9]	27.3 [24.6; 30.7]
≤18.5 or ≥25.0 kg/m^2^	193,260 (65.7)	112,273 (63.1)	45,853 (68.1)	35,134 (71.7)
Had seen a doctor for mental health problem, *n* (%)	98,707 (33.5)	53,182 (29.9)	23,825 (35.4)	21,700 (44.3)
Poor diet *, *n* (%)	140,761 (47.8)	82,386 (46.3)	32,944 (48.9)	25,431 (51.9)
Physical activity ≤3000 MET-minutes/week, *n* (%)	201,616 (68.5)	121,790 (68.5)	44,863 (66.6)	34,963 (71.3)
HLS, *n* (%)				
High (4–5 points)	30,500 (10.4)	21,194 (11.9)	6175 (9.2)	3131 (6.4)
Medium (2–3 points)	142,355 (48.4)	89,655 (50.4)	31,964 (47.4)	20,736 (42.3)
Low (0–1 points)	121,360 (41.2)	66,984 (37.7)	29,228 (43.4)	25,148 (51.3)

Abbreviations: CKD, chronic kidney disease; BMI, body mass index; HLS, healthy lifestyle score; MET, Metabolic Equivalent Task. * Poor diet was defined as not meeting at least four of the seven food groups: fruits ≥ 3 servings/day, vegetables ≥ 3 servings/day, fish ≥ 2 servings/week, whole grains ≥ 3 servings/day, refined grain ≤ 1.5 servings/day, unprocessed meats ≤ 1.5 servings/week, and processed meats ≤1 servings/week. Data are expressed as median (interquartile range) or proportion *n* (%).

**Table 2:** Associations of sleep patterns and HLS with CKD risk.

Characteristic	Case/Total	HR (95% CI)		
		Model 1 *	Model 2 ^†^	Model 3 ^‡^
Sleep patterns				
Healthy sleep pattern	9317/175,312	1.00 (reference)	1.00 (reference)	1.00 (reference)
Intermediate sleep pattern	7466/112,536	1.22 (1.18–1.26)	1.15 (1.11–1.19)	1.13 (1.09–1.17)
Poor sleep pattern	574/6367	1.33 (1.28–1.38)	1.23 (1.18–1.28)	1.18 (1.14–1.23)
*p*-value for trend ^‖^	--	<0.001	<0.001	<0.001
HLS				
High HLS	1937/50,402	1.00 (reference)	1.00 (reference)	1.00 (reference)
Medium HLS	10,177/177,808	1.60 (1.49–1.71)	1.45 (1.35–1.55)	1.44 (1.34–1.54)
Low HLS	5243/66,005	2.26 (2.11–2.41)	1.88 (1.76–2.01)	1.85 (1.73–1.98)
*p*-value for trend ^‖^	--	<0.001	<0.001	<0.001

Abbreviations: BMI, body mass index; CI, confidence interval; CKD, chronic kidney disease; HLS, healthy lifestyle score; HR, hazard ratio. * Adjusted for age (continuous), sex (male or female), and ethnicity (white or nonwhite). ^†^ Adjusted for covariates in model 1 plus Townsend deprivation index (low, intermediate, or high), education (higher degree, any school degree, vocational qualifications, or unknown), household income (less than 51,999, greater than 52,000, or unknown), hypertension (yes or no), and diabetes (yes or no). ^‡^ Mutually adjusted for sleep patterns or HLS levels as appropriate. ^‖^ *p*-value for trend calculated treating the sleep patterns or HLS as a continuous variable.

**Table 3:** Associations between sleep patterns and CKD stratified by HLS.

Characteristic	High HLS		Medium HLS		Low HLS	
	HR (95% CI)	*p*-Value	HR (95% CI)	*p*-Value	HR (95% CI)	*p*-Value
Healthy sleep pattern	1.00 (reference)	reference	1.00 (reference)	reference	1.00 (reference)	reference
Intermediate sleep pattern	1.08 (0.93–1.26)	0.315	1.10 (1.04–1.16)	<0.001	1.17 (1.11–1.23)	<0.001
Poor sleep pattern	1.07 (0.88–1.31)	0.486	1.12 (1.05–1.19)	<0.001	1.23 (1.17–1.30)	<0.001
*p*-value for trend ^‖^	--	0.314	--	<0.001	--	<0.001

Abbreviations: CI, confidence interval; CKD, chronic kidney disease; HLS, healthy lifestyle score; HR, hazard ratio. All results were calculated adjusted by age (continuous), sex (male or female), ethnicity (white or nonwhite), Townsend deprivation index (low, intermediate, or high), education (higher degree, any school degree, vocational qualifications, or unknown), household income (less than 51,999, greater than 52,000, or unknown), hypertension (yes or no), and diabetes (yes or no). ^‖^ *p* value for trend calculated treating the sleep patterns as a continuous variable.

## Data Availability

All data used in this study are publicly accessible from the UK Biobank via their standard data access procedure at https://www.ukbiobank.ac.uk (accessed on 25 May 2024).

## Supplementary Materials

The following supporting information can be downloaded at https://www.mdpi.com/article/10.3390/nu16234238/s1: Figure S1: Cumulative incidence of CKD across different HLS levels in participants with healthy sleep pattern. Figure S2: Cumulative incidence of CKD across different HLS levels in participants with intermediate sleep pattern. Figure S3: Cumulative incidence of CKD across different HLS levels in participants with poor sleep pattern. Figure S4: Joint association of sleep patterns and HLS with CKD risk. (Dyslipidemia is added to the covariates). Table S1: ICD 10 code used in disease definition. Table S2: Definitions and field ID used for each component of sleep patterns. Table S3: Associations of five metrics of sleep patterns with CKD risk. Table S4: Definitions and field ID used for each component of HLS. Table S5: Associations of five metrics of HLS with CKD risk. Table S6: Baseline characteristics of study participants by HLS levels. Table S7: Population attributable fraction of sleep patterns and HLS. Table S8: Associations of CKD with five metrics of sleep patterns within HLS. Table S9: Associations of CKD with sleep patterns within five metrics of HLS. Table S10: Joint association of sleep patterns and HLS with CKD risk. Table S11: Risk of incident CKD according to sleep patterns and HLS stratified by sociodemographic variables. Table S12: Associations between sleep patterns and CKD stratified by HLS. (After excluding participants who developed CKD events within two years of follow-up, *N* = 291,496.) Table S13: Joint association of sleep patterns and HLS with CKD risk. (After excluding participants who developed CKD events within two years of follow-up, *N* = 291,496.) Table S14: Associations between sleep patterns and CKD stratified by HLS. (Sleep patterns were constructed using sleep factors that remained significant after multivariate adjustment.) Table S15: Joint association of sleep patterns and HLS with CKD risk. (Sleep patterns were constructed using sleep factors that remained significant after multivariate adjustment.) Table S16: Associations between sleep patterns and CKD stratified by HLS. (Sleep patterns were constructed using sleep factors that remained significant after multivariate adjustment, and participants who developed CKD events within 2 years of follow-up were excluded, *N* = 291,496.) Table S17: Joint association of sleep patterns and HLS with CKD risk. (Sleep patterns were constructed using sleep factors that remained significant after multivariate adjustment, and participants who developed CKD events within 2 years of follow-up were excluded, *N* = 291,496.) Table S18: Associations between sleep patterns and CKD stratified by HLS. (Dyslipidemia was added to the covariates.)

## Abbreviations

ACR	albumin–creatinine ratio
AP	attributable proportion due to interaction
BMI	body mass index
CI	confidence interval
CKD	chronic kidney disease
eGFR	estimated glomerular filtration rate
HbA1c	glycated hemoglobin
HLS	healthy lifestyle score
HR	hazard ratio
ICD10	the International Classification of Diseases, 10th revision
IQR	interquartile range
MET	Metabolic Equivalent Task
PAF	population attributable fraction
RERI	relative excess risk due to interaction
T2D	type 2 diabetes
TDI	Townsend deprivation index
