# Supplementary material for: Healthy Lifestyle Behaviors Attenuate the Effect of Poor Sleep Patterns on Chronic Kidney Disease Risk: A Prospective Study from the UK Biobank
Source: Nutrients. 2024 Dec 8;16(23):4238. doi: 10.3390/nu16234238 (PMC11644827; doi:10.3390/nu16234238)
Supplement: Supplementary file 1 [file nutrients-16-04238-s001.zip › nutrients-3335294-supplementary.pdf]

Supplemental Materials

Supplemental Methods

Supplemental Table S1. ICD 10 code used in disease definition.

Supplemental Table S2. Definitions and field ID used for each component of sleep patterns.

Supplemental Table S3. Associations of five metrics of sleep patterns with CKD risk.

Supplemental Table S4. Definitions and field ID used for each component of HLS.

Supplemental Table S5. Associations of five metrics of HLS with CKD risk.

Supplemental Table S6. Baseline characteristics of study participants by HLS levels.

Supplemental Table S7. Population attributable fraction of sleep patterns and HLS.

Supplemental Table S8. Associations of CKD with five metrics of sleep patterns within HLS.

Supplemental Table S9. Associations of CKD with sleep patterns within five metrics of HLS.

Supplemental Table S10. Joint association of sleep patterns and HLS with CKD risk.

Supplemental Table S11. Risk of incident CKD according to sleep patterns and HLS stratified by sociodemographic variables.

Supplemental Table S12. Associations between sleep patterns and CKD stratified by HLS. (After excluding participants who developed CKD events within two years of follow-up, *N*= 291,496.)

Supplemental Table S13. Joint association of sleep patterns and HLS with CKD risk. (After excluding participants who developed CKD events within two years of follow-up, *N*= 291,496).

Supplemental Table S14. Associations between sleep patterns and CKD stratified by HLS. (Sleep patterns were constructed using sleep factors that remained significant after multivariate adjustment.)

Supplemental Table S15. Joint association of sleep patterns and HLS with CKD risk. (Sleep patterns were constructed using sleep factors that remained significant after multivariate adjustment.)

Supplemental Table S16. Associations between sleep patterns and CKD stratified by HLS. (Sleep patterns were constructed using sleep factors that remained significant after multivariate adjustment and participants who developed CKD events within 2 years of follow-up were excluded, *N*= 291,496.)

Supplemental Table S17. Joint association of sleep patterns and HLS with CKD risk. (Sleep patterns were constructed using sleep factors that remained significant after multivariate adjustment and participants who developed CKD events within 2 years of follow-up were excluded, *N*= 291,496.)

Supplemental Table S18. Associations between sleep patterns and CKD stratified by HLS. (Dyslipidemia was added to the covariates.)

Supplemental Figure S1. Cumulative incidence of CKD across different HLS levels in participants with healthy sleep pattern.

Supplemental Figure S2. Cumulative incidence of CKD across different HLS levels in participants with intermediate sleep pattern.

Supplemental Figure S3. Cumulative incidence of CKD across different HLS levels in participants with poor sleep pattern.

Supplemental Figure S4. Joint association of sleep patterns and HLS with CKD risk. (Dyslipidemia is added to the covariates).

Supplemental Methods

Ascertainment of Chronic Kidney Disease

For females, eGFR is calculated as $144 \times (serum\ creatinine/62)^{-0.329} \times (0.993)^{age}$ when serum creatinine is ≤ 62 mg/dL, and $144 \times (serum\ creatinine/62)^{-1.209} \times (0.993)^{age}$ when it exceeds 62 mg/dL. For males, the formula is $141 \times (serum\ creatinine/80)^{-0.411} \times (0.993)^{age}$ if serum creatinine is ≤ 80 mg/dL, and $141 \times (serum\ creatinine/80)^{-1.209} \times (0.993)^{age}$ if it exceeds 80 mg/dL[1].

Covariates

For the classification of ethnicity, participants identified as British, White and Black Caribbean, Indian, and Caribbean were categorized as "white". All other major categories and their respective subcategories, including Mixed, Asian or Asian British, Black or Black British, Chinese, and Other ethnic group, were categorized as "nonwhite".

Townsend deprivation index was calculated before participants joined the UK Biobank and was based on the preceding national census data, with each participant assigned a score corresponding to the postcode of their home dwelling.

Household income was assessed using the question, "What is the average total income before tax received by your HOUSEHOLD?" Participants could choose from the following response categories: "Less than 18,000," "18,000 to 30,999," "31,000 to 51,999," "52,000 to 100,000," "Greater than 100,000," "Do not know," and "Prefer not to answer." For the purposes of our analysis, these responses were reclassified into three categories: "Less than 51,999," "Greater than 52,000," and "Unknown" (which included "Do not know" and "Prefer not to answer").

Education (Qualifications) were assessed using the question, "Which of the following qualifications do you have?" The response categories included: "College or University degree", "A levels/AS levels or equivalent", "O levels/GCSEs or equivalent", "CSEs or equivalent", "NVQ or HND or HNC or equivalent", "Other professional qualifications eg: nursing, teaching", "None of the above", and "Prefer not to answer". These responses were reclassified into four categories: "College or University degree" was defined as "Higher Degree", "Other professional qualifications (e.g., nursing, teaching)" were defined as "Vocational Qualifications", and "None of the above" or "Prefer not to answer" were grouped into the "Unknown" category. All other responses were classified as "Any School Degree".

Supplemental Table S1. ICD 10 code used in disease definition.

**Diseases**	**ICD 10**
Chronic nephritic syndrome	N03
Isolated proteinuria with specified morphological lesion	N06
Glomerular disorders in diseases classified elsewhere	N08
Chronic tubulo-interstitial nephritis	N11
Tubulo-interstitial nephritis, not specified as acute or chronic	N12
Obstructive and reflux uropathy	N13
Drug- and heavy-metal-induced tubulo-interstitial and tubular conditions	N14
Other renal tubulo-interstitial diseases	N15
Renal tubulo-interstitial disorders in diseases classified elsewhere	N16
Chronic renal failure	N18
Unspecified renal failure	N19
Calculus of kidney and ureter	N20
Calculus of lower urinary tract	N21

Abbreviations: ICD10, the International Classification of Diseases, 10th revision.

Supplemental Table S2. Definitions and field ID used for each component of sleep patterns.

Item	Description	Definitions of ideal/poor sleep behavior	Field ID
Sleep duration	Sleep duration of the participants was assessed by asking the question "About how many hours sleep do you get in every 24 hours? (please include naps)"	7-8 hours were defined as the ideal length of sleep duration, and the remaining answers were defined as poor.	1160
Chronotype	Chronotype was assessed by asking the question "Do you consider yourself to be?", with the response categories: "Definitely a 'morning' person", "More a 'morning' than 'evening' person", "More an 'evening' than 'morning' person", "Definitely an 'evening' person", "Do not know", and "Prefer not to answer".	Answers with "Definitely a 'morning' person" and "More a 'morning' than 'evening' person" were defined as "Morning chronotype"; answers with "Definitely a 'evening' person" and "More an 'evening' than 'morning' person" were defined as "Evening chronotype".	1180
Insomnia	Insomnia was assessed by asking the question "Do you have trouble falling asleep at night or do you wake up in the middle of the night?", with the response categories: "Never/rarely", "Sometimes", "Usually", and "Prefer not to answer".	Answer with "Never/rarely", "Sometimes" was defined as "Free of insomnia", and "Usually" was defined as "Insomnia".	1200
Snoring	Snoring was assessed by asking the question "Does your partner or a close relative or friend complain about your snoring?", with the response categories: "Yes", "No", "Do not know", and "Prefer not to answer".	Answer with "No" was defined as "No snoring", and "Yes" was defined as "Snoring".	1210
Daytime dozing	Daytime dozing was assessed by asking the question "How likely are you to doze off or fall asleep during the daytime when you do not mean to?", with the response categories: "Never/rarely", "Sometimes", "Often", "Do not know", "Prefer not to answer", and "All of the time".	Answer with "Never/rarely", "Sometimes" was defined as "No frequent daytime dozing", and "Often" was defined as "Daytime dozing".	1220

Supplemental Table S3. Associations of five metrics of sleep patterns with CKD risk.

Characteristic	Case/Total	HR (95% CI)				
		Model 1*	*p*-value	Model 2†	*p*-value	β
Poor sleep duration (<7h/day or >8h/day)	6171/89,316	1.25 (1.21-1.29)	< 0.001	1.13 (1.10-1.17)	< 0.001	0.125
Evening chronotype	6317/109,178	1.06 (1.03-1.10)	< 0.001	1.02 (0.99-1.06)	0.157	0.023
Insomnia	13,449/218,327	1.15 (1.11-1.19)	<0.001	1.06 (1.02-1.10)	0.003	0.056
Snoring	7133/108,219	1.11 (1.08-1.14)	<0.001	1.01 (0.98-1.04)	0.554	0.009
Daytime dozing	4939/65,545	1.19 (1.15-1.23)	<0.001	1.10 (1.06-1.13)	<0.001	0.092

Abbreviations: CI, confidence interval; CKD, chronic kidney disease; HLS, healthy lifestyle score; HR, hazard ratio.

* Adjusted for age (continuous), sex (male or female) and ethnicity (white or non white).

† Adjusted for covariates in model 1 plus Townsend deprivation index (low, intermediate or high), education (higher degree, any school degree, vocational qualifications or unknown), household income (less than 51,999, greater than 52,000 or unknown), hypertension (yes or no), diabetes (yes or no) and HLS (high, medium or low), with other sleep factors being mutually adjusted as appropriate.

Supplemental Table S4. Definitions and field ID used for each component of HLS.

Item	Definitions of ideal/poor lifestyle behavior	Conversion of units	Field ID
Smoking status	Only never smoker was defined as ideal.	--	20116
Physical activity	Metabolic equivalent ≥ 3000 MET-minutes/week was defined as ideal, and the remaining METs were defined as poor physical activity.	Walking MET-minutes/week<br>= 3.3 * walking minutes * walking days;<br>Moderate MET-minutes/week<br>= 4.0 * moderate-intensity activity minutes * moderate days;<br>Vigorous MET-minutes/week<br>= 8.0 * vigorous-intensity activity minutes * vigorous-intensity days.	914, 904, 894, 884, 874, 864
Diet	At least 4 of the following 7 food groups was defined as ideal diet:<br>1. Fruits: ≥3 servings/day;<br>2. Vegetables: ≥3 servings/day;<br>3. Fish: ≥2 servings/week;<br>4. Whole grains: ≥3 servings/day;<br>5. Refined grain: ≤1.5 servings/day;<br>6. Unprocessed meats: ≤1.5 servings/week;<br>7. Processed meats: ≤1 servings/week.	One serving of every food group equals to:<br>1. 1 piece of fresh fruit or 5 pieces of dried fruit;<br>2. 3 heaped tablespoons;<br>3. Once/week fish;<br>4. 1 slice of whole-grain bread or 1 cup of whole-grain cereal;<br>5. 1 slice of bread or 1 bowl of cereal;<br>6. Once/week unprocessed meats;<br>7. Once/week processed meats.	1309, 1319, 1289, 1299, 1329, 1339, 1438, 1448, 1458, 1468, 1369, 1379, 1389, 1349
BMI	BMI value here is constructed from height and weight measured during the initial Assessment Centre visit. It was calculated as the weight (kg) divided by height squared (m^2). 18.5 ≤ BMI <25.0 kg/m^2 was defined as ideal BMI.	--	21001
Mental health	Never seen a doctor for nerves, anxiety, tension or depression was defined as ideal mental health.	--	2090, 2100

Abbreviations: BMI, body mass index; HLS, healthy lifestyle scores; MET, Metabolic Equivalent Task.

Supplemental Table S5. Associations of five metrics of HLS with CKD risk.

Characteristic[ξ]	Case/Total	HR (95% CI)				
		Model 1*	*p*-value	Model 2†	*p*-value	β
Poor physical activity	11,959/201,616	1.09 (1.05-1.12)	<0.001	1.08 (1.04-1.11)	<0.001	0.073
Poor smoking status	9048/131,769	1.21 (1.17-1.25)	<0.001	1.10 (1.07-1.14)	<0.001	0.099
Poor diet	9211/140,761	1.29 (1.25-1.33)	<0.001	1.19 (1.15-1.22)	<0.001	0.171
Poor BMI	13,418/193,260	1.63 (1.57-1.69)	<0.001	1.37 (1.32-1.42)	<0.001	0.312
Poor mental health	6301/98,707	1.26 (1.22-1.30)	<0.001	1.18 (1.15-1.22)	<0.001	0.169

Abbreviations: BMI, body mass index; CI, confidence interval; CKD, chronic kidney disease; HLS, healthy lifestyle score; HR, hazard ratio.

ξ Metabolic equivalent <3000 MET-minutes/week was defined as poor physical activity, current or previous smoking was defined as Poor smoking status, not meeting at least four of the seven food groups was defined as poor diet, BMI⩽18.5 or ⩾25.0 kg/m^2 was defined as poor BMI, Had seen a doctor for mental health problem was defined as poor mental health.

* Adjusted for age (continuous), sex (male or female) and ethnicity (white or non white).

† Adjusted for covariates in model 1 plus Townsend deprivation index (low, intermediate or high), education (higher degree, any school degree, vocational qualifications or unknown), household income (less than 51,999, greater than 52,000 or unknown), hypertension (yes or no), diabetes (yes or no) and sleep patterns (healthy, intermediate or poor), with other lifestyle behaviors being mutually adjusted as appropriate.

Supplemental Table S6. Baseline characteristics of study participants by HLS levels.

Characteristic	Total	High HLS	Medium HLS	Low HLS
Participants, *N* (%)	294,215 (100.0)	30,500 (10.4)	142,355 (48.4)	121,360 (41.2)
CKD, *n* (%)	17,357 (5.9)	977 (3.2)	7484 (5.3)	8896 (7.3)
Age, *n* (%)				
Continuous, years	57.0 [49.0;63.0]	57.0 [49.0;62.0]	57.0 [49.0;63.0]	57.0 [49.0;62.0]
<65	245,466 (83.4)	25,494 (83.6)	118,090 (83.0)	101,882 (84.0)
≥65	48,749 (16.6)	5006 (16.4)	24,265 (17.0)	19,478 (16.0)
Female, *n* (%)	156,587 (53.2)	21,036 (69.0)	79,205 (55.6)	56,346 (46.4)
White Ethnicity, *n* (%)	268,305 (91.2)	27,769 (91.0)	129,426 (90.9)	111,110 (91.6)
Townsend deprivation index, *n* (%)				
Low (Quantile1)	104,136 (35.4)	12,231 (40.1)	51,723 (36.3)	40,182 (33.1)
Intermediate (Quantile2)	99,813 (33.9)	10,538 (34.6)	48,826 (34.3)	40,449 (33.3)
High (Quantile3)	89,915 (30.6)	7700 (25.2)	41,650 (29.3)	40,565 (33.4)
Education, *n* (%)				
Higher degree	107,320 (36.5)	14,528 (47.6)	54,679 (38.4)	38,113 (31.4)
Any school degree	132,650 (45.1)	11,865 (38.9)	62,741 (44.1)	58,044 (47.8)
Vocational qualifications	14,842 (5.0)	1518 (5.0)	7249 (5.1)	6075 (5.0)
Household income Less than 51,999, *n* (%)	185,417 (63.0)	17,087 (56.0)	88,069 (61.9)	80,261 (66.1)

Diabetes, *n* (%)	13,625 (4.6)	440 (1.4)	5382 (3.8)	7803 (6.4)
Hypertension, *n* (%)	145,773 (49.5)	10,760 (35.3)	66,663 (46.8)	68,350 (56.3)
Sleep duration, *n* (%)				
Continuous, hours/day	7.17 (1.1)	7.20 (0.9)	7.17 (1.0)	7.15 (1.1)
Long (>8h)	20,872 (7.1)	1527 (5.0)	9153 (6.4)	10,192 (8.4)
Normal (7-8h)	204,899 (69.6)	23,160 (75.9)	10,1527 (71.3)	80,212 (66.1)
Short (<7h)	68,444 (23.3)	5813 (19.1)	31,675 (22.3)	30,956 (25.5)
Chronotype, *n* (%)				
Morning	78,441 (26.7)	8854 (29.0)	39,473 (27.7)	30,114 (24.8)
More morning than evening	106,596 (36.2)	12,315 (40.4)	52,883 (37.1)	41,398 (34.1)
More evening than morning	82,963 (28.2)	7485 (24.5)	38,770 (27.2)	36,708 (30.2)
Evening	26,215 (8.91)	1846 (6.05)	11,229 (7.89)	13,140 (10.8)
Insomnia, *n* (%)				
Never/rarely	75,888 (25.8)	9111 (29.9)	38,417 (27.0)	28,360 (23.4)
Sometimes	139,984 (47.6)	14,834 (48.6)	68,306 (48.0)	56,844 (46.8)
Usually	78,343 (26.6)	6555 (21.5)	35,632 (25.0)	36,156 (29.8)
Snoring, *n* (%)	108,219 (36.8)	6469 (21.2)	47,008 (33.0)	54,742 (45.1)
Daytime dozing, *n* (%)				
Never/rarely	228,670 (77.7)	24,971 (81.9)	112,534 (79.1)	91,165 (75.1)

Sometimes	58,366 (19.8)	5077 (16.6)	26,827 (18.8)	26,462 (21.8)
Often/always	7179 (2.44)	452 (1.48)	2994 (2.10)	3733 (3.08)
Sleep pattern, *n* (%)				
Healthy (4-5 points)	177,833 (60.4)	21,194 (69.5)	89,655 (63.0)	66,984 (55.2)
Intermediate (2-3 points)	67,367 (22.9)	6175 (20.2)	31,964 (22.5)	29,228 (24.1)
Poor (0-1 points)	49,015 (16.7)	3131 (10.3)	20,736 (14.6)	25,148 (20.7)

Abbreviations: CKD, chronic kidney disease; HLS, healthy lifestyle score. Data are expressed as median (interquartile range) or proportion n (%).

Supplemental Table S7. Population attributable fraction of sleep patterns and HLS.

Component	PAF (95%CI), %
Sleep duration	3.90 (2.88-4.92)
Chronotype	0.90 (-0.17-1.97)
Insomnia	4.47 (1.92-7.03)
Snoring	1.18 (-0.02-2.38)
Daytime dozing	2.39 (1.51-3.26)
**Healthy sleep pattern**	**5.53 (4.29-6.77)**
Physical activity	4.38 (2.33-6.43)
Smoking status	3.73 (2.28-5.18)
Diet	7.59 (6.15-9.02)
BMI	20.15 (18.00-22.30)
Mental health	5.53 (4.50-6.56)
**Healthy lifestyle**	**33.96 (30.05-37.86)**
**Healthy lifestyle and healthy sleep pattern**	**36.06 (31.35-40.77)**

Abbreviations: BMI, body mass index; CI, confidence interval; HLS, healthy lifestyle score; PAF, population attributable fraction.

PAFs and 95% CIs were calculated adjusting for age (continuous), sex (male or female) and ethnicity (white or non white), Townsend deprivation index (low, intermediate or high), education (higher degree, any school degree, vocational qualifications or unknown), household income (less than 51,999, greater than 52,000 or unknown), hypertension (yes or no) and diabetes (yes or no).

Supplemental Table S8. Associations of CKD with five metrics of sleep patterns within HLS.

Characteristic	High HLS		Medium HLS		Low HLS		*p* for interaction
	HR (95% CI)	*p*-value	HR (95% CI)	*p*-value	HR (95% CI)	*p*-value	
**Sleep duration**							
Normal(7-8h)	1.00 (reference)	reference	1.00 (reference)	reference	1.00 (reference)	reference	0.022
Poor	1.03 (0.89-1.19)	0.667	1.10 (1.05-1.15)	< 0.001	1.18 (1.13-1.23)	< 0.001	
**Chronotype**							0.583
Morning	1.00 (reference)	reference	1.00 (reference)	reference	1.00 (reference)	reference	
Evening	1.01 (0.87-1.16)	0.941	1.04 (0.99-1.09)	0.087	1.01 (0.97-1.05)	0.664	
**Insomnia**							0.723
No	1.00 (reference)	reference	1.00 (reference)	reference	1.00 (reference)	reference	
Yes	1.11 (0.96-1.29)	0.160	1.06 (1.00-1.12)	0.043	1.06 (1.00-1.11)	0.043	
**Snoring**							0.546
No	1.00 (reference)	reference	1.00 (reference)	reference	1.00 (reference)	reference	
Yes	0.94 (0.81-1.10)	0.429	0.99 (0.95-1.04)	0.779	1.04 (1.00-1.09)	0.048	
**Daytime dozing**							0.143
No	1.00 (reference)	reference	1.00 (reference)	reference	1.00 (reference)	reference	
Yes	1.01 (0.86-1.18)	0.916	1.16 (1.10-1.22)	< 0.001	1.06 (1.01-1.11)	0.012	

Abbreviations: CI, confidence interval; CKD, chronic kidney disease; HLS, healthy lifestyle score; HR, hazard ratio.

Adjusted for age (continuous), sex (male or female), ethnicity (white or non white), Townsend deprivation index (low, intermediate or high), education (higher degree, any school degree, vocational qualifications or unknown), household income (less than 51,999, greater than 52,000 or unknown), hypertension (yes or no), diabetes (yes or no) with other sleep factors being mutually adjusted as appropriate.

Supplemental Table S9. Associations of CKD with sleep patterns within five metrics of HLS.

Characteristic	Healthy sleep pattern		Intermediate sleep pattern		Poor sleep pattern		*p* for interaction
	HR (95% CI)	*p*-value	HR (95% CI)	*p*-value	HR (95% CI)	*p*-value	
**Physical activity**							0.427
Ideal	1.00 (reference)	reference	1.07 (1.01-1.14)	0.022	1.22 (1.13-1.32)	< 0.001	
Poor	1.00 (reference)	reference	1.10 (1.05-1.14)	< 0.001	1.25 (1.18-1.31)	< 0.001	
**Smoking status**							0.566
Ideal	1.00 (reference)	reference	1.10 (1.05-1.15)	< 0.001	1.26 (1.18-1.34)	< 0.001	
Poor	1.00 (reference)	reference	1.08 (1.03-1.13)	< 0.001	1.22 (1.15-1.29)	< 0.001	
**Diet**							0.019
Ideal	1.00 (reference)	reference	1.06 (1.01-1.11)	0.018	1.18 (1.11-1.25)	< 0.001	
Poor	1.00 (reference)	reference	1.12 (1.07-1.17)	< 0.001	1.30 (1.22-1.37)	< 0.001	
**BMI**							0.085
Ideal	1.00 (reference)	reference	1.09 (1.02-1.17)	0.014	1.13 (1.03-1.24)	0.010	
Poor	1.00 (reference)	reference	1.09 (1.05-1.13)	< 0.001	1.27 (1.21-1.33)	< 0.001	
**Mental health**							0.645
Ideal	1.00 (reference)	reference	1.09 (1.04-1.13)	< 0.001	1.24 (1.17-1.31)	< 0.001	
Poor	1.00 (reference)	reference	1.09 (1.03-1.16)	0.002	1.24 (1.16-1.32)	< 0.001	

Abbreviations: CI, confidence interval; CKD, chronic kidney disease; HLS, healthy lifestyle score; HR, hazard ratio.

Adjusted for age (continuous), sex (male or female), ethnicity (white or non white), Townsend deprivation index (low, intermediate or high), education (higher degree, any school degree, vocational qualifications or unknown), household income (less than 51,999, greater than 52,000 or unknown), hypertension (yes or no), diabetes (yes or no) with other lifestyle behaviors being mutually adjusted as appropriate.

Supplemental Table S10. Joint association of sleep patterns and HLS with CKD risk.

Characteristic	HLS (HR, 95% CI)			HLS (RERI*, 95% CI)		HLS (AP*, 95% CI)		*p* for interaction
	High HLS	Medium HLS	Low HLS	Medium HLS	Low HLS	Medium HLS	Low HLS	
**Sleep patterns**								
**Healthy**	1.00	1.43 (1.31-1.55)	1.77 (1.63-1.93)					
**Intermediate**	1.08 (0.93-1.26)	1.57 (1.44-1.72)	2.06 (1.89-2.26)	0.05 (-0.04-0.14)	0.13 (0.09-0.18)	0.04 (-0.04-0.11)	0.07 (0.03-0.11)	0.026
**Poor**	1.07 (0.88-1.31)	1.60 (1.45-1.76)	2.19 (2.00-2.40)	0.07 (-0.04-0.19)	0.18 (0.14-0.23)	0.05 (-0.05-0.15)	0.11 (0.05-0.17)	

Abbreviations: CI, confidence interval; CKD, chronic kidney disease; HLS, healthy lifestyle score; HR, hazard ratio; RERI, relative excess risk due to interaction; AP, attributable proportion due to interaction.

Adjusted for age (continuous), sex (male or female) and ethnicity (white or non white), Townsend deprivation index (low, intermediate or high), education (higher degree, any school degree, vocational qualifications or unknown), household income (less than 51,999, greater than 52,000 or unknown), hypertension (yes or no) and diabetes (yes or no).

*The estimates of RERI and AP were calculated based on the reference group with healthy sleep pattern and high HLS.

Supplemental Table S11. Risk of incident CKD according to sleep patterns and HLS stratified by sociodemographic variables.

Subgroup	Age, HR (95% CI)		Sex, HR (95% CI)		Hypertension, HR (95% CI)		Diabetes, HR (95% CI)	
	<65	>=65	Female	Male	Hypertension	Non-Hypertension	Diabetes	Non-Diabetes
**High HLS**								
Healthy sleep pattern	reference	reference	reference	reference	reference	reference	reference	reference
Intermediate sleep pattern	1.22 (1.01-1.47)	0.93 (0.72-1.21)	1.06 (0.88-1.29)	1.11 (0.87-1.42)	1.20 (0.97-1.49)	0.98 (0.78-1.22)	1.07 (0.49-2.35)	1.08 (0.92-1.26)
Poor sleep pattern	1.22 (0.96-1.56)	0.92 (0.65-1.31)	1.03 (0.80-1.33)	1.13 (0.82-1.56)	1.07 (0.80-1.43)	1.09 (0.82-1.43)	0.84 (0.29-2.46)	1.08 (0.88-1.33)
**Medium HLS**								
Healthy sleep pattern	1.47 (1.33-1.63)	1.33 (1.15-1.52)	1.33 (1.20-1.49)	1.52 (1.33-1.74)	1.60 (1.42-1.81)	1.27 (1.13-1.43)	1.63 (1.05-2.54)	1.42 (1.30-1.54)
Intermediate sleep pattern	1.64 (1.46-1.83)	1.45 (1.24-1.68)	1.52 (1.35-1.71)	1.63 (1.41-1.88)	1.75 (1.54-1.99)	1.42 (1.24-1.62)	1.88 (1.20-2.95)	1.55 (1.41-1.70)
Poor sleep pattern	1.73 (1.53-1.94)	1.43 (1.22-1.68)	1.55 (1.37-1.76)	1.64 (1.41-1.91)	1.74 (1.53-1.99)	1.50 (1.30-1.72)	2.07 (1.32-3.26)	1.56 (1.41-1.72)
**Low HLS**								
Healthy sleep pattern	1.77 (1.59-1.96)	1.69 (1.46-1.94)	1.74 (1.56-1.94)	1.81 (1.58-2.06)	1.98 (1.75-2.23)	1.60 (1.42-1.80)	2.04 (1.32-3.16)	1.76 (1.61-1.92)
Intermediate sleep pattern	2.11 (1.89-2.35)	1.87 (1.61-2.17)	2.14 (1.91-2.41)	2.03 (1.76-2.33)	2.33 (2.06-2.64)	1.81 (1.59-2.07)	2.41 (1.55-3.74)	2.05 (1.87-2.24)
Poor sleep pattern	2.28 (2.04-2.55)	1.87 (1.61-2.19)	2.27 (2.02-2.55)	2.13 (1.85-2.45)	2.45 (2.17-2.78)	1.96 (1.71-2.25)	2.49 (1.61-3.87)	2.18 (1.99-2.39)
***p* for interaction**	0.034		<0.001		0.295		0.766	

Abbreviations: CI, confidence interval; CKD, chronic kidney disease; HLS, healthy lifestyle score; HR, hazard ratio.

Adjusted for age (continuous), sex (male or female), ethnicity (white or non white), Townsend deprivation index (low, intermediate or high), education (higher degree, any school degree, vocational qualifications or unknown), household income (less than 51,999, greater than 52,000 or unknown), hypertension (yes or no) and diabetes (yes or no) as appropriate.

Supplemental Table S12. Associations between sleep patterns and CKD stratified by HLS. (After excluding participants who developed CKD events within two years of follow-up, *N*= 291,496.)

Characteristic	High HLS		Medium HLS		Low HLS	
	HR (95% CI)	*p*-value	HR (95% CI)	*p*-value	HR (95% CI)	*p*-value
Healthy sleep pattern	1.00 (reference)	reference	1.00 (reference)	reference	1.00 (reference)	reference
Intermediate sleep pattern	1.06 (0.91-1.25)	0.456	1.11 (1.05-1.18)	< 0.001	1.14 (1.08-1.20)	< 0.001
Poor sleep pattern	1.10 (0.90-1.36)	0.354	1.13 (1.06-1.21)	< 0.001	1.23 (1.16-1.30)	< 0.001
p-value for trend‖	--	0.279	--	< 0.001	--	< 0.001

Abbreviations: CI, confidence interval; CKD, chronic kidney disease; HLS, healthy lifestyle score; HR, hazard ratio.

All results were calculated adjusted by age (continuous), sex (male or female) and ethnicity (white or non white), Townsend deprivation index (low, intermediate or high), education (higher degree, any school degree, vocational qualifications or unknown), household income (less than 51,999, greater than 52,000 or unknown), hypertension (yes or no) and diabetes (yes or no).

‖ *P*-value for trend calculated treating the sleep patterns as a continuous variable.

Supplemental Table S13. Joint association of sleep patterns and HLS with CKD risk. (After excluding participants who developed CKD events within two years of follow-up, *N*= 291,496.)

Characteristic	HLS (HR, 95% CI)			HLS (RERI*, 95% CI)		HLS (AP*, 95% CI)		*p* for interaction
	High HLS	Medium HLS	Low HLS	Medium HLS	Low HLS	Medium HLS	Low HLS	
**Sleep patterns**								
**Healthy**	1.00	1.41 (1.29-1.54)	1.77 (1.62-1.93)					
**Intermediate**	1.06 (0.90-1.25)	1.57 (1.42-1.72)	2.03 (1.85-2.22)	0.07 (-0.03-0.17)	0.12 (0.07-0.16)	0.05 (-0.04-0.14)	0.07 (0.02-0.11)	0.083
**Poor**	1.10 (0.89-1.35)	1.59 (1.44-1.76)	2.19 (1.99-2.40)	0.07 (-0.05-0.19)	0.18 (0.13-0.22)	0.05 (-0.05-0.15)	0.10 (0.04-0.16)	

Abbreviations: CI, confidence interval; CKD, chronic kidney disease; HLS, healthy lifestyle score; HR, hazard ratio; RERI, relative excess risk due to interaction; AP, attributable proportion due to interaction.

Adjusted for age (continuous), sex (male or female) and ethnicity (white or non white), Townsend deprivation index (low, intermediate or high), education (higher degree, any school degree, vocational qualifications or unknown), household income (less than 51,999, greater than 52,000 or unknown), hypertension (yes or no) and diabetes (yes or no).

*The estimates of RERI and AP were calculated based on the reference group with healthy sleep pattern and high HLS.

Supplemental Table S14. Associations between sleep patterns and CKD stratified by HLS. (Sleep patterns were constructed using sleep factors that remained significant after multivariate adjustment.)

**Characteristic**	**High HLS**		**Medium HLS**		**Low HLS**	
	**HR (95% CI)**	***p*-value**	**HR (95% CI)**	***p*-value**	**HR (95% CI)**	***p*-value**
Healthy sleep pattern	1.00 (reference)	reference	1.00 (reference)	reference	1.00 (reference)	reference
Intermediate sleep pattern	1.04 (0.88-1.25)	0.627	1.09 (1.02-1.16)	0.008	1.15 (1.09-1.22)	< 0.001
Poor sleep pattern	1.03 (0.89-1.20)	0.660	1.12 (1.06-1.18)	< 0.001	1.22 (1.16-1.28)	< 0.001
p-value for trend‖	--	0.614	--	< 0.001	--	< 0.001

Abbreviations: CI, confidence interval; CKD, chronic kidney disease; HLS, healthy lifestyle score; HR, hazard ratio.

All results were calculated adjusted by age (continuous), sex (male or female) and ethnicity (white or non white), Townsend deprivation index (low, intermediate or high), education (higher degree, any school degree, vocational qualifications or unknown), household income (less than 51,999, greater than 52,000 or unknown), hypertension (yes or no) and diabetes (yes or no).

‖ *P* value for trend calculated treating the sleep patterns as a continuous variable.

Supplemental Table S15. Joint association of sleep patterns and HLS with CKD risk. (Sleep patterns were constructed using sleep factors that remained significant after multivariate adjustment.)

Characteristic	HLS (HR, 95% CI)			HLS (RERI*, 95% CI)		HLS (AP*, 95% CI)		*p* for interaction
	High HLS	Medium HLS	Low HLS	Medium HLS	Low HLS	Medium HLS	Low HLS	
**Sleep patterns**								
**Healthy**	1.00	1.41 (1.29-1.53)	1.74 (1.59-1.90)					
**Intermediate**	1.04 (0.87-1.24)	1.53 (1.39-1.69)	1.99 (1.81-2.19)	0.06 (-0.05-0.17)	0.12 (0.07-0.18)	0.05 (-0.05-0.14)	0.07 (0.02-0.13)	0.004
**Poor**	1.03 (0.89-1.20)	1.56 (1.43-1.71)	2.13 (1.95-2.33)	0.09 (-0.01-0.18)	0.17 (0.13-0.22)	0.06 (-0.03-0.16)	0.11 (0.05-0.17)	

Abbreviations: CI, confidence interval; CKD, chronic kidney disease; HLS, healthy lifestyle score; HR, hazard ratio; RERI, relative excess risk due to interaction; AP, attributable proportion due to interaction.

Adjusted for age (continuous), sex (male or female) and ethnicity (white or non white), Townsend deprivation index (low, intermediate or high), education (higher degree, any school degree, vocational qualifications or unknown), household income (less than 51,999, greater than 52,000 or unknown), hypertension (yes or no) and diabetes (yes or no).

*The estimates of RERI and AP were calculated based on the reference group with healthy sleep pattern and high HLS.

Supplemental Table S16. Associations between sleep patterns and CKD stratified by HLS. (Sleep patterns were constructed using sleep factors that remained significant after multivariate adjustment and participants who developed CKD events within 2 years of follow-up were excluded, *N*= 291,496.)

Characteristic	High HLS		Medium HLS		Low HLS	
	HR (95% CI)	*p*-value	HR (95% CI)	*p*-value	HR (95% CI)	*p*-value
Healthy sleep pattern	1.00 (reference)	reference	1.00 (reference)	reference	1.00 (reference)	reference
Intermediate sleep pattern	1.05 (0.87-1.26)	0.609	1.11 (1.04-1.18)	0.002	1.13 (1.06-1.20)	< 0.001
Poor sleep pattern	1.03 (0.88-1.21)	0.719	1.12 (1.06-1.18)	< 0.001	1.21 (1.16-1.28)	< 0.001
p-value for trend‖	--	0.664	--	< 0.001	--	< 0.001

Abbreviations: CI, confidence interval; CKD, chronic kidney disease; HLS, healthy lifestyle score; HR, hazard ratio.

All results were calculated adjusted by age (continuous), sex (male or female) and ethnicity (white or non white), Townsend deprivation index (low, intermediate or high), education (higher degree, any school degree, vocational qualifications or unknown), household income (less than 51,999, greater than 52,000 or unknown), hypertension (yes or no) and diabetes (yes or no).

‖ *P* value for trend calculated treating the sleep patterns as a continuous variable.

Supplemental Table S17. Joint association of sleep patterns and HLS with CKD risk. (Sleep patterns were constructed using sleep factors that remained significant after multivariate adjustment and participants who developed CKD events within 2 years of follow-up were excluded, *N*= 291,496.)

Characteristic	HLS (HR, 95% CI)			HLS (RERI*, 95% CI)		HLS (AP*, 95% CI)		*p* for interaction
	High HLS	Medium HLS	Low HLS	Medium HLS	Low HLS	Medium HLS	Low HLS	
**Sleep patterns**								
**Healthy**	1.00	1.39 (1.27-1.52)	1.74 (1.58-1.90)					0.007
**Intermediate**	1.05 (0.87-1.26)	1.54 (1.39-1.71)	1.96 (1.77-2.16)	0.08 (-0.04-0.19)	0.11 (0.05-0.16)	0.05 (-0.05-0.16)	0.06 (0.01-0.11)	
**Poor**	1.03 (0.88-1.20)	1.54 (1.40-1.70)	2.12 (1.93-2.33)	0.09 (-0.01-0.19)	0.17 (0.13-0.22)	0.07 (-0.03-0.17)	0.11 (0.05-0.18)	

Abbreviations: CI, confidence interval; CKD, chronic kidney disease; HLS, healthy lifestyle score; HR, hazard ratio; RERI, relative excess risk due to interaction; AP, attributable proportion due to interaction.

Adjusted for age (continuous), sex (male or female) and ethnicity (white or non white), Townsend deprivation index (low, intermediate or high), education (higher degree, any school degree, vocational qualifications or unknown), household income (less than 51,999, greater than 52,000 or unknown), hypertension (yes or no) and diabetes (yes or no).

*The estimates of RERI and AP were calculated based on the reference group with healthy sleep pattern and high HLS.

Supplemental Table S18. Associations between sleep patterns and CKD stratified by HLS. (Dyslipidemia is added to the covariates.)

Characteristic	High HLS		Medium HLS		Low HLS	
	HR (95% CI)	*p*-value	HR (95% CI)	*p*-value	HR (95% CI)	*p*-value
Healthy sleep pattern	1.00 (reference)	reference	1.00 (reference)	reference	1.00 (reference)	reference
Intermediate sleep pattern	1.08 (0.92-1.25)	0.344	1.10 (1.04-1.16)	0.001	1.16 (1.10-1.22)	< 0.001
Poor sleep pattern	1.08 (0.88-1.32)	0.471	1.11 (1.04-1.18)	0.001	1.22 (1.16-1.28)	< 0.001
p-value for trend[‖]	--	0.317	--	< 0.001	--	< 0.001

Abbreviations: CI, confidence interval; CKD, chronic kidney disease; HLS, healthy lifestyle score; HR, hazard ratio.

All results were calculated adjusted by age (continuous), sex (male or female) and ethnicity (white or non white), Townsend deprivation index (low, intermediate or high), education (higher degree, any school degree, vocational qualifications or unknown), household income (less than 51,999, greater than 52,000 or unknown), hypertension (yes or no), diabetes (yes or no), dyslipidemia (yes or no).

Dyslipidemia was determined from medication for cholesterol lowering at baseline.

‖ *P* value for trend calculated treating the sleep patterns as a continuous variable.

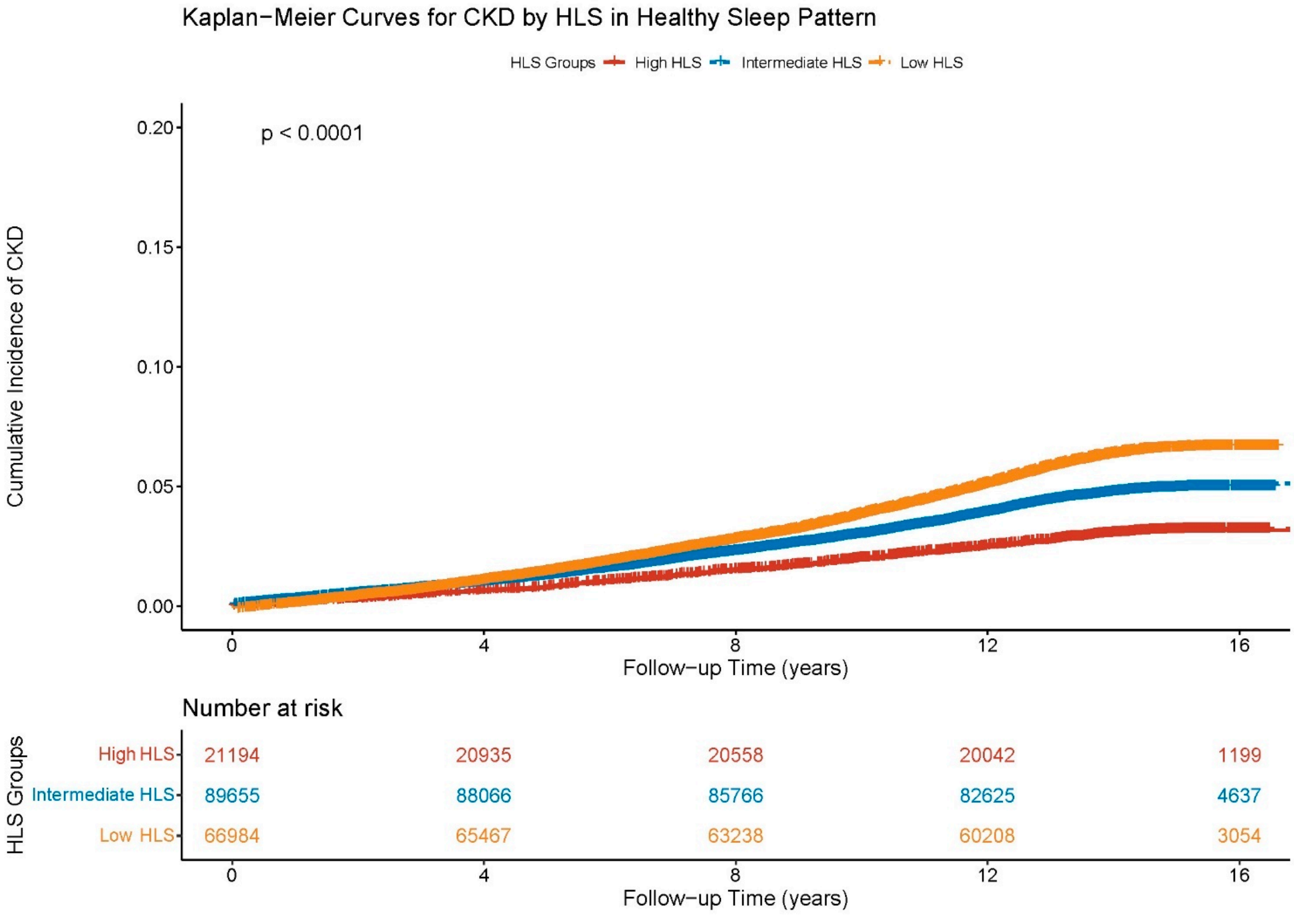

Supplemental Figure S1. Cumulative incidence of CKD across different HLS levels in participants with healthy sleep pattern.

Abbreviations: CKD, chronic kidney disease; HLS, healthy lifestyle score.

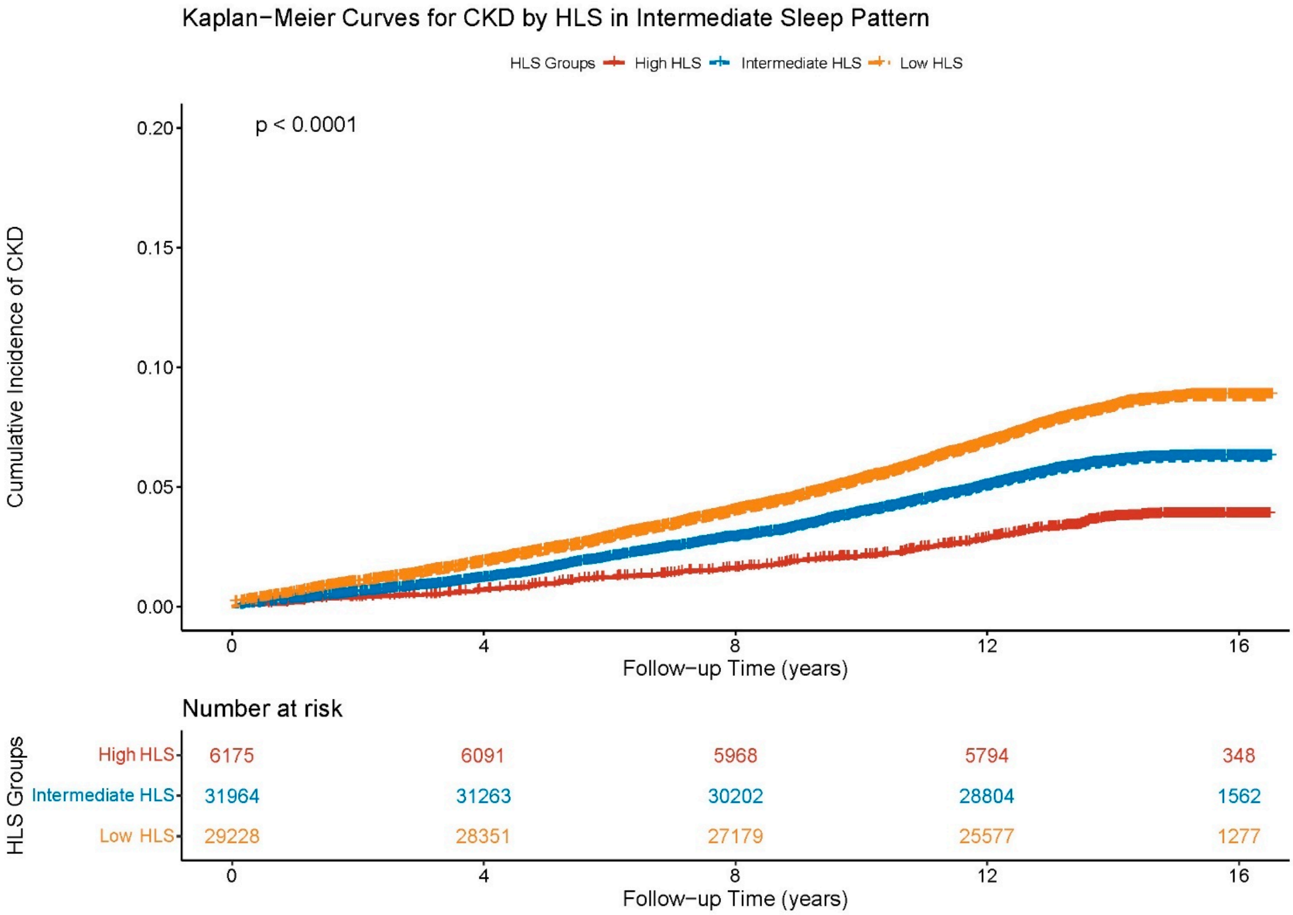

Supplemental Figure S2. Cumulative incidence of CKD across different HLS levels in participants with intermediate sleep pattern.

Abbreviations: CKD, chronic kidney disease; HLS, healthy lifestyle score.

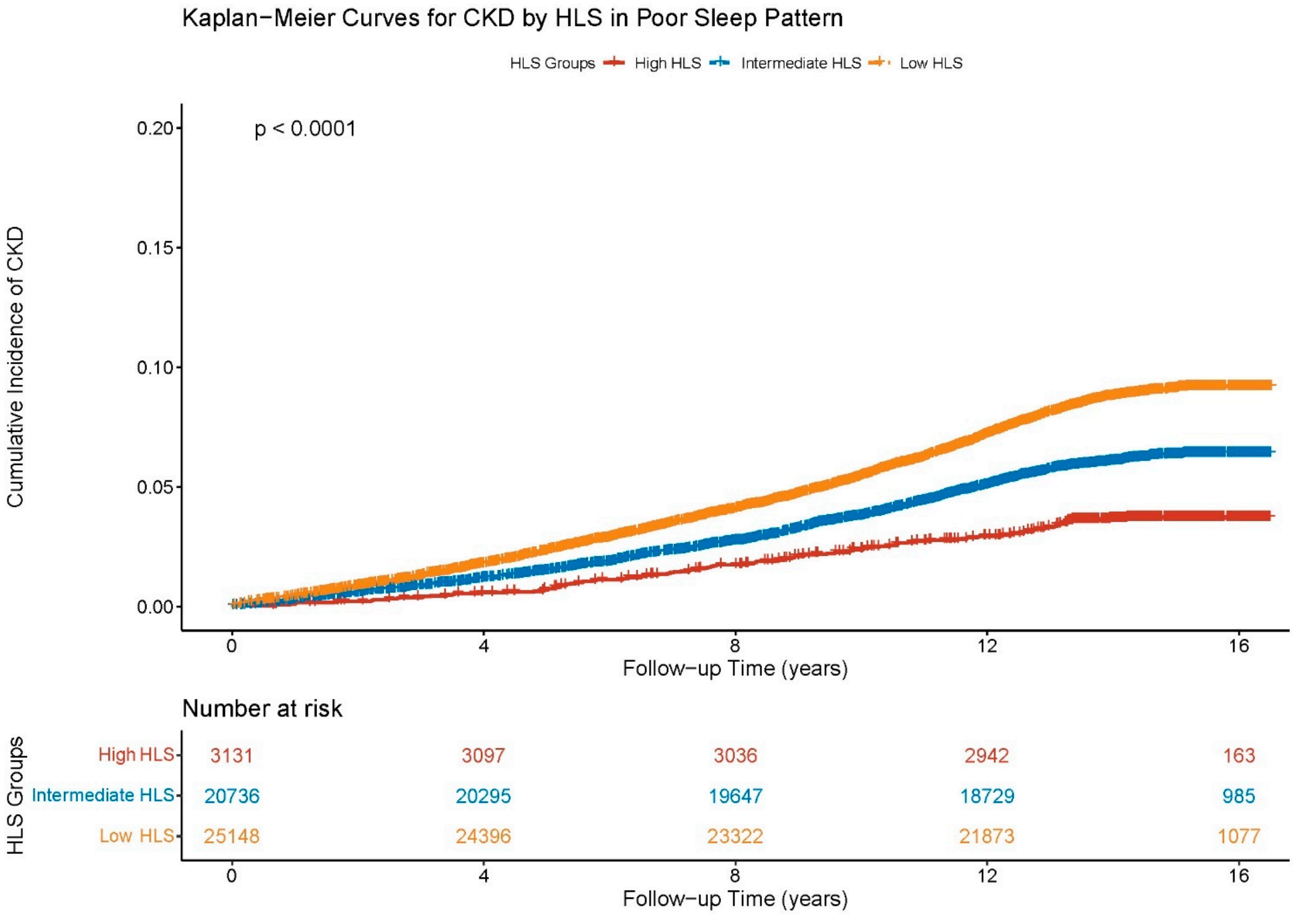

Supplemental Figure S3. Cumulative incidence of CKD across different HLS levels in participants with poor sleep pattern.

Abbreviations: CKD, chronic kidney disease; HLS, healthy lifestyle score.

Subgroup	No. of patients	Cases	HR(95% CI)	*P* value
**High HLS**				
Healthy sleep pattern	21,194	639	Reference	Reference
Intermediate sleep pattern	6175	225	1.08 (0.92−1.25)	0.345
Poor sleep pattern	3131	113	1.07 (0.88−1.31)	0.490
**Medium HLS**				
Healthy sleep pattern	89,655	4380	1.41 (1.30−1.53)	<0.001
Intermediate sleep pattern	31,964	1858	1.55 (1.42−1.70)	<0.001
Poor sleep pattern	20,736	1246	1.57 (1.43−1.73)	<0.001
**Low HLS**				
Healthy sleep pattern	66,984	4360	1.74 (1.60−1.89)	<0.001
Intermediate sleep pattern	29,228	2367	2.01 (1.84−2.20)	<0.001
Poor sleep pattern	25,148	2169	2.12 (1.94−2.32)	<0.001

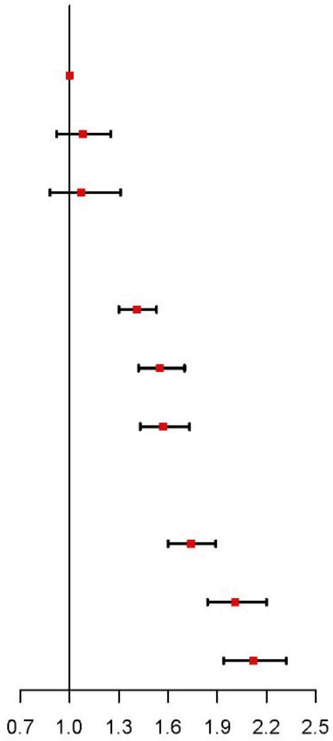

Supplemental Figure S4. Joint association of sleep patterns and HLS with CKD risk. (Dyslipidemia is added to the covariates.) CKD = chronic kidney disease; HLS = healthy lifestyle score; CI = confidence interval; HR = hazard ratio. Dyslipidemia was determined from medication for cholesterol lowering at baseline.
